# Strategies for increasing impact, engagement, and accessibility in HIV prevention programs: suggestions from women in urban high HIV burden counties in the Eastern United States (HPTN 064)

**DOI:** 10.1186/s12889-020-09426-6

**Published:** 2020-09-03

**Authors:** Jasmine A. Abrams, Michelle Odlum, Emily Tillett, Danielle Haley, Jessica Justman, Sally Hodder, Linda Vo, Ann O’Leary, Paula M. Frew, the HIV Prevention Trials Network 064 (HTPN) Study Team

**Affiliations:** 1 Department of Community Health Sciences, Boston University School of Public Health, 801 Massachusetts Avenue (Crosstown Center), Rm 434, Boston, MA 02118 USA; 2 Center for Interdisciplinary Research on AIDS, Yale University School of Public Health, New Haven, CT USA; 3 Columbia University School of Nursing, New York, NY USA; 4 Department of Psychology, University of Maryland, Baltimore County, Baltimore, MD USA; 5 Mailman School of Public Health, Columbia University, New York, NY USA; 6 West Virginia Clinical and Translational Science Institute, West Virginia University, Morgantown, WV USA; 7 Department of Medicine, Division of Infectious Diseases, Emory University School of Medicine, Atlanta, GA USA; 8 Division of HIV/AIDS Prevention, Centers for Disease Control and Prevention, Atlanta, GA USA; 9 School of Public Health, University of Nevada, Las Vegas, NV USA; 10 Population Health & Health Equity Initiative, University of Nevada, Las Vegas, NV USA

**Keywords:** Sexual health, Implementation science, HIV hotspots, Biobehavioral intervention, PrEP

## Abstract

**Background:**

Merely having the tools to end HIV is insufficient. Effectively ending the epidemic necessitates addressing barriers that impede engagement in biomedical and behavioral prevention and wide scale implementation and utilization of existing interventions. This qualitative study identifies suggestions for increasing access to, engagement in, and impact of HIV prevention among women living in cities in high HIV burden counties in the eastern US.

**Methods:**

Data analyzed for the current study were collected via a qualitative sub-study within the HIV Prevention Trials Network Study 064 (HPTN 064), a multisite observational cohort study designed to estimate HIV incidence among women residing in communities with elevated HIV prevalence who also reported personal or partner characteristics associated with increased risk of HIV acquisition. Focus group and interview participants in the qualitative sub-study (*N* = 288) were from four cities in the eastern US.

**Results:**

Thematic analyses revealed four themes describing women’s most frequently stated ideas for improving prevention efforts: 1) Promote Multilevel Empowerment, 2) Create Engaging Program Content, 3) Build “Market Demand”, and 4) Ensure Accessibility. We conducted additional analyses to identify contradictory patterns in the data, which revealed an additional three themes: 1) Address Structural Risk Factors, 2) Increase Engagement via Pleasure Promotion, 3) Expand Awareness of and Access to Prevention Resources.

**Conclusions:**

Findings may be useful for enhancing women’s engagement in and uptake of behavioral and biomedical HIV prevention resources, improving policy, and addressing multilevel risk factors.

**Trial registration:**

Clinicaltrials.gov: NCT00995176, prospectively registered.

## Background

The United States (US)’ National AIDS Strategy prioritizes promotion of HIV prevention service utilization and provision of community-based services to improve access to and awareness of these services in US communities [1, 2]. The resulting development and implementation of targeted and tailored public health programs, awareness campaigns, and interventions have contributed to major accomplishments in HIV prevention, including decreases in sexual and perinatal transmission as well as increases in span and quality of life for people living with HIV/AIDS (PLWHA) [3–6]. As indicated in the national *Ending the Epidemic* initiative [3], such accomplishments suggest that the knowledge, tools, and resources needed to end the HIV/AIDS epidemic currently exist. Despite these successes, HIV remains a glaring example of health inequity in the US, frequently concentrating in socially disadvantaged groups with epidemic rates only in certain types of communities – communities with high numbers of marginalized individuals (including, but not limited to, non-Hispanic Black, Latinx, low income, gay, and transgender communities) [7–9]. Furthermore, although rates of diagnoses have declined among women, nearly every hour a woman tests positive for HIV [10]. Moreover, racial/ethnic disparities persist. For example, continuing a 30 year trend, Black women account for the majority of women living with HIV/AIDS, making Black women 15 and 5 times more likely to contract HIV than White women and Latinas, respectively [10].

Among women, there are numerous multilevel barriers to engagement in existing HIV prevention approaches, including poverty, incarceration, unemployment, distrust of providers and locations where HIV prevention services are offered, displeasures associated with safer sex practices, intimate partner violence, challenges related to gendered power, cultural issues, stigma associated with HIV and women’s sexual behavior, and a lack of intervention strategies that address risk across multiple levels [11–17]. Thus, merely having the tools to end HIV is insufficient. Effectively ending the epidemic necessitates addressing barriers that impede engagement in biomedical and behavioral prevention and wide scale implementation and utilization of existing interventions [18].

As such, science that seeks to advance health equity in HIV requires an understanding of what is needed from the perspective of the target population and how that information can be translated to practice to promote equity [19–21]. Furthermore, garnering key insights from the target population can help ensure the development of culturally relevant interventions that can contribute to greater engagement and uptake of prevention resources compared to approaches solely developed by researchers and practitioners [21]. To this end, the goal of the current study was to identify suggestions for HIV prevention programs among women residing in cities with a high burden of HIV and poverty, with an emphasis on acquiring the perspectives of Black women given their increased risk for acquiring HIV. This study offers findings that can be utilized to enhance current interventions and provides strategies for addressing barriers to successful implementation of behavioral and biomedical interventions among women at risk for acquiring HIV.

## Methods

Data analyzed for the current study were collected via a qualitative sub-study within the HIV Prevention Trials Network Study 064 (HPTN 064), a multisite observational cohort study designed to estimate HIV incidence among women residing in communities with elevated HIV prevalence who also reported personal or partner characteristics associated with increased risk of HIV acquisition. Ethical approvals for the study were obtained from Institutional Review Boards at collaborating institutions at each study site. Details of the methods of the HPTN 064 Women’s HIV SeroIncidence Study have been previously described in other publications [22, 23].

### Participant recruitment and data collection

Individuals who self-identified as women, ages 18–44 years, who resided in census tracts or zip codes ranking in the top 30th percentile of HIV prevalence in urban cities (Atlanta, Baltimore, New York City, Newark, Raleigh-Durham, and Washington, DC) were enrolled in the overall study using venue-based and quota sampling [22]. Upon enrolling in the larger study, a subset of 120 women were first selected for interviews using purposive and quota sampling among women with odd participant identification numbers. Upon completing the interviews, purposive sampling was used to identify additional women eligible to participate in focus groups. Women selected for interviews were not selected as participants for focus groups.

Between May 2009 and July 2010, a total of 31 focus groups were conducted across the sites. Participants in the qualitative sub-study (*N* = 288) were from four cities in the eastern US: 1) Bronx, New York; 2) Washington, DC; 3) Raleigh, North Carolina; and 4) Atlanta, Georgia. Focus groups consisted of 8–10 women and were stratified by age and race/ethnicity – a strategy used to group individuals with similar life experiences [24].

The overarching goal of both the interviews and focus groups was to explore and contextualize multilevel risk factors for HIV acquisition as well as strategies for reducing women’s risk for HIV. Written consent was obtained and focus groups and interviews were conducted in private settings (e.g., private rooms at the study sites) to ensure privacy and confidentiality. To protect their identities, participants in focus groups were asked to identify themselves each time they spoke using a pseudonym. All focus groups and interviews were led by young to middle aged female facilitators and the ethnicity of facilitators was matched to focus group participants as often as possible.

Focus group and interview participants were compensated for their involvement (between $30 - $50 USD, with variability between sites based on cost of living differences). All focus groups and interviews were recorded and transcribed by trained research team members. Transcripts were reviewed for accuracy and anonymized before being uploaded into a qualitative data analysis software program, NVivo 8.0 [25, 26].

### Data analysis

Group discussions and interviews were guided by a series of open-ended questions aimed at better understanding women’s risk for HIV and suggestions for risk reduction. Parts of the interview guide were developed for the current study and have been published elsewhere [11]. Data for the current study were generated from the following question: “If someone gave you money to design a health program to promote women’s sexual health, and keep them from getting HIV and other STIs, what would you like to make sure the program included?” Participants were probed on program content, implementation, and marketing. Data were analyzed using an interpretative paradigmatic approach, which seeks to understand phenomena by identifying and constructing knowledge based on inter-subjectively shared meanings among study participants. In this way, the interpretive paradigm maximizes subjectivity and can provide marginalized groups the opportunity to share, in their own words, their experiences and opinions [27, 28]. Such data can be useful in supporting and challenging existing practices and policies in healthcare [29, 30].

#### Inductive thematic analysis

Thematic analysis of transcribed data occurred in three phases: 1) Structural Coding, 2) Preliminary Analysis and Member Checking, and 3) Advanced Systematic Analysis [31].

#### Structural coding and preliminary analysis

Structural coding, Phase 1, was utilized to develop a preliminary coding scheme (i.e., codebook) by reading all of the transcripts and categorizing text associated with questions in the data collection guide. During Phase 2, members of the research team reviewed and analyzed data for a subset of the structural codes, defined preliminary themes, and developed corresponding conceptual frameworks within and across structural codes. Given the large volume of data, during Phase 3 we selected a random subsample of 30% of the interview transcripts and approximately 50% of the focus group transcripts for advanced coding and subsequent analysis. Achievement of data saturation (i.e., the point in the coding process when new codes emerge) for the coding scheme and thematic content determined the sizes of these subsamples [32].

#### Advanced systematic thematic analysis

The advanced analysis began with initial coding, where we reviewed transcribed data to create codes and categories and to identify emerging themes. Next, during axial coding, we coded larger segments of text, noted potential associations among codes, and developed descriptive subcodes and categories. Finally, during selective coding, we reviewed the code categories and individually coded information relating to that category (hierarchical coding). Throughout this iterative process, codes and themes were constantly compared and refined until achievement of thematic saturation [31, 32].

#### Disconfirming case analysis

In addition to the inductive thematic analysis, we conducted disconfirming case analysis, based on established and validated analytic methods for qualitative research, to identify contradictory patterns in the data. This process, also referred to as negative or deviant case analysis in the literature, is used to enhance the quality and rigor of qualitative analysis [33]. The process involves identifying and discussing data that differ from primary themes (i.e., themes generated from prevalent codes) by identifying less common themes in the data in addition to alternative explanations for primary themes [34, 35]. This technique assists researchers with improving and refining analyses by allowing researchers to explain the majority of the data and capture counter narratives in comparison to solely discussing data that reflects the views of the most participants [33–35]. Such an approach can be particularly useful in studies with sensitive topics where social desirability may be high among participants (e.g., discussing sex). In the current study, contrary cases were identified by examining codes developed during the thematic analysis that 1) differed from codes and themes in the thematic analysis and 2) were mentioned at least five times across groups and interviews.

### Scientific rigor and Intercoder reliability

Several strategies were used to strengthen scientific rigor in this study. For example, we utilized open-ended questions to foster authenticity of the data [36]. Clarification techniques were employed to ensure understanding of participant remarks. In addition, we employed iterative processes with multiple coders, peer examination, and inductive data analysis with Nvivo 8, a qualitative software package [25]. Further, intercoder reliability (ICR) was assessed. Two analysists independently coded all transcripts that were selected for advanced systematic analysis. The final ICR was calculated manually using traditional calculation methods based on percent agreement and disagreement generated by the NVivo 8 ICR function. The analysts reviewed, resolved, and amended any coding discrepancies for transcripts with a Kappa below 0.80. Our final ICR reflects a combined average Kappa score of 0.91 for the focus groups and 0.92 for the interviews, indicative of a high level of coding consistency [37].

## Results

### Characteristics of the sample

Among participants enrolled in the qualitative sub-study (*N* = 288), 86% were Black, 67% non-partnered, 59% had a household income of $20,000 or less, and 2% had an HIV seropositive diagnosis. Of sub-study participants reporting vaginal or anal sex within the last six months, the majority of participants reported not using a condom during their last instance of vaginal or anal intercourse. As an additional level of rigor, we ran descriptive statistics, t-tests, and chi-square tests between our sub-sample (*N* = 288) and the overall study population (*N* = 2099) to confirm that no meaningful differences existed in demographic characteristics between our subgroup participants and the overall study participants. Additional demographic information for participants can be found in Table 1. For ease of presentation, participants are categorized as Black or non-Black in the table.

**Table 1:** Demographics and behavioral characteristics of interview and focus group participants and overall HPTN 064 cohort

Characteristic	Qualitative data (interview or focus group)	Overall data	P (t-test or chi-squared)
Number of Women	288	2099	
Age			
Median	27	29	
25th, 75th %tile	23, 36	23, 38	
Race			0.9685
Non-Black	41/288 (14%)	297/2099 (14%)	
Black	247/288 (86%)	1802/2099 (86%)	
Education			0.4486
Less than High School	100/288 (35%)	777/2099 (37%)	
> = High School	188/288 (65%)	1322/2099 (63%)	
Marital status			0.1438
Missing	8/288 (3%)	51/2099 (2%)	
Married	30/288 (10%)	159/2099 (8%)	
Not married, living together	56/288 (19%)	479/2099 (23%)	
Non-partnered	194/288 (67%)	1410/2099 (67%)	
Household Income			0.4714
$10,000 or less	134/288 (47%)	933/2099 (44%)	
$10,001 to $20,000	34/288 (12%)	225/2099 (11%)	
$20,001 or More	31/288 (11%)	197/2099 (9%)	
Refused to answer/Don’t know	89/288 (31%)	744/2099 (35%)	
Number of male partners in last 6 months			
Median	2	2	
25th, 75th %tile	1, 3	1, 3	
Condom used at last vaginal intercourse			0.8562
NA	1/288 (< 1%)	7/2099 (< 1%)	
Yes	55/288 (19%)	376/2099 (18%)	
No	230/288 (80%)	1698/2099 (81%)	
Don’t Know	2/288 (1%)	18/2099 (1%)	
Any anal sex in past 6 months			0.7409
Missing	1/288 (< 1%)	5/2099 (< 1%)	
No	175/288 (61%)	1298/2099 (62%)	
Yes	112/288 (39%)	796/2099 (38%)	
Condom used at last anal intercourse			0.2712
Yes	23/112 (21%)	143/796 (18%)	
No	89/112 (79%)	637/796 (80%)	
Don’t know	0/112 (0%)	16/796 (2%)	
Missing	0/112 (0%)	0/796 (0%)	
Exchange sex in past 6 months			0.3703
Missing	2/288 (1%)	21/2099 (1%)	
No	187/288 (65%)	1302/2099 (62%)	
Yes	99/288 (34%)	776/2099 (37%)	
Own concurrent partnership in past 6 months			0.6938
Missing	1/288 (< 1%)	9/2099 (< 1%)	
No	177/288 (61%)	1314/2099 (63%)	
Yes	110/288 (38%)	776/2099 (37%)	
HIV seropositive diagnosis			0.5836
No	283/288 (98%)	2071/2099 (99%)	
Yes	5/288 (2%)	28/2099 (1%)	

### Thematic analysis results

Thematic analysis revealed four main themes related to HIV prevention programming and services: 1) Promote Multilevel Empowerment (Subthemes: Personal Empowerment, Relational Empowerment, and Multilevel Empowerment), 2) Create Engaging Program Content, 3) Build “Market Demand” (Subtheme: Branding), and 4) Ensure Accessibility. Detailed descriptions of each theme and related subthemes are presented in the following paragraphs and a summary of the results are depicted in Table 2.

**Table 2:** Summary of thematic and disconfirming case analyses

Themes	Subthemes	Example Quotes
**Thematic Analysis Results**		
Promote Multilevel Empowerment	Personal Empowerment Relational Empowerment Multilevel Empowerment	“So, she needs education first [then] empowerment and self-esteem reinforcement … All that and prevention needs to be available.” *Black woman, Raleigh*
Create Engaging Program Content		“…Half the time somebody hands me a brochure, I’m not gonna read it. I don’t care what it’s got on it… I want something hands on.” *Black woman, Raleigh*
Build “Market Demand”	Branding	[Programs need] “to make somebody’s mouth water for it.” *Black woman, Bronx*
Ensure Accessibility		“I would just make everything accessible … Because it’ll make a person come on in. Design a program *with* folks – to bring women in.” *Black woman, Washington DC*
**Disconfirming Case Analysis Results**		
Address Structural Risk Factors	Reduce Poverty as a Risk Factor Employ Policy to Mandate Prevention Participation	“Most times, it’s finances, and women … take higher risks to get what has to get paid, so there [needs to be] more programs out to help them pay their bills, and feed their children, and clothe them.” *Black woman, Bronx*
Expand Awareness of and Access to Prevention Resources		“The free condoms, even though they’re free, they’re not available in every corner store. It’s not in walking distance… So I would say just, promote more condoms.” *Hispanic woman, Bronx*
Increase Engagement via Pleasure Promotion		“My point is … there should be … ways to make safe sex fun… knowing that you’re talking about your safety or whatever, maybe you find different ways to make it fun.” *Hispanic woman, Bronx*

#### Promote multilevel empowerment

Across groups, participants discussed the need for prevention programs to focus on empowering women by providing them with the confidence and skills needed to make self-determined choices that represent their interests in safer sex. Collectively, participants suggested that such empowerment should be addressed at individual, interpersonal, and systemic levels.

##### Personal empowerment

Based on group discussions, programs should include strategies that empower women as individuals to ensure women have “the ability to make these choices for themselves and [for them to] know that they’re making the decision that they feel is best for them” (*Black woman, Raleigh*). At the individual level, this concept was reflective of the idea that programs should teach women strategies for “building their self-esteem up [and] being confident” (*Hispanic woman, Bronx*). Participants contextualized the need for individual empowerment via improvement of self-esteem by suggesting that women with lower self-esteem may be more likely to look to sex or men as a coping strategy for avoiding or mitigating negative self-perceptions. Thus, participants expressed that it is imperative for HIV prevention programs to include content focused on improving mental health and self-esteem, especially given the ability of the latter to impact sexual health decision making.

I would focus on … self-esteem, the inner woman, because at the end of the day I think that’s what drives a lot of women who are promiscuous into being promiscuous is that low self-esteem … a lot of what we feel inside and what we’re going through inside has a lot to do with … what men we choose, how often we switch sexual partners…

*Black woman, Raleigh*

In addition, participants explained that negative self-perceptions influence women to defer to desires of and ingratiate themselves with male partners for condomless sex, even if women desire or plan to use condoms. Explaining how low self-esteem may contribute to increased sexual risk behaviors and demonstrating the importance of addressing it in prevention, a Black woman from Atlanta simply stated that programs “… would have to include … self-esteem.” Participants believed that addressing self-esteem would make women less likely to rely on men or sex to elevate their esteem and would encourage women to recognize that their desires are just as important and valuable as those of their partners.

##### Relational empowerment

Implicit in participant comments on the essentiality of addressing self-esteem to reduce sexual risk behavior was an acknowledgement that risk behavior does not occur in isolation and that women must also be interpersonally empowered in order to engage in protective sexual health decision making. In this regard, participants called for programs to include strategies that empower women to address barriers to safer sex that arise with others. To promote empowerment at an interpersonal level, participants believed programs should enhance women’s sexual communication skills. Women discussed the need for programs to train women to be confident and bold when discussing safer sex with partners so that they could insist on use of protection. In one rapid focus group exchange between several women, participants explicitly stated women needed training in:

“Communication skills”; “With partners and stuff …”; “What kind of questions to ask, especially what kind of questions to ask. How to say, how to be more…”; “Assertive”; “That’s the word I was looking for! Be more assertive…”; “And more firm about it….”; “Not to offend the person.”

*Five Hispanic women, Bronx*

In another focus group exchange participants discussed how individual empowerment, in the form of high self-esteem and confidence, could help facilitate interpersonal empowerment in the context of sexual communication with partners. They also described how such confidence could be increased via group discussions with other women.

“Confidence, a lot of women need confidence to be able to talk about things like that with their partners.”; “Building their self-esteem up, being confident.”; “Exactly.”; “You know? Women, you know, they can just talk about it and get that boost [of confidence].”; “Yeah, from each other.”

*Three Hispanic Participants, Bronx*

Discussions regarding the need for programs to train women in sexual communication skills were at times contextualized with descriptions of gender imbalances that perpetuated women’s loss of power and agency in being able to negotiate safer sex with partners.

There’s so many women available and there’s so many men that’s not available. So many men incarcerated. So many men in mental facilities. So many men at war. They’re limited and [women] don’t think that they could find something maybe half as good as what they have …So they’d rather settle.

*Black woman, Bronx*

Participants suggested that given the low ratio of men to women and women’s subsequent loss of power, women often tolerate less than desirable relationship conditions. Focusing specifically on sexual relations, they emphasized that if a woman wants to keep a man she may be more apprehensive about having a discussion on safer sex. Moreover, if she does raise the topic, she has to be strategic in how she approaches the subject so as not to lose her partner in the process.

Because a lot of times… women [would] rather not have that uncomfortable conversation and risk getting her man upset because of that - because she wants to use the condom and he might not want to use it. So she don’t want to ruffle the feathers or whatever. So she’ll avoid it and risk it all ...

*Hispanic woman, Bronx*

Participants acknowledged the difficulty many women experience in trying to negotiate safer sex practices and simultaneously highlighted this as a needed area of focus for prevention programming.

In addition to being empowered in relationships with partners, women discussed the importance of being empowered to have conversations about sex with their children. As one participant stated, “Some women need to have help with how to present… those type[s] of topics and ideas to their partners … Or how to talk to their kids or their teenagers about sex and being protected …” (*Black woman, Raleigh*). In many groups, women noted that parent-child sexual education needed to begin earlier in childhood given that sex is initiated at younger ages in their communities. Though many participants discussed the need to have these conversations with teenagers, numerous participants explicitly stated that they should also have “sex talks” with children in elementary school.

They say you not supposed to get sex ed until you thirteen or you in middle school but it’s kids in elementary school having sex but they not, well they think they too young to talk about it. For real, for real I think that I should have known about sex when I was in fourth grade. (*Black woman, Washington DC*)

Many women passionately discussed the importance of providing training for women to teach their children and other young people in their communities about HIV prevention. Participants emphasized that it was important for young people to have access to sexual health information in schools given that “abstinence only” education would be insufficient because many young people are already sexually active. “It would be nice for kids to be abstinent, but I don’t think that’s reasonable to think it’s going to happen” (*Black woman, Bronx*). In light of the lack of comprehensive sex education in schools, participants noted the role of parents in ensuring that their children had enough information to make protective sexual health decisions. “You can’t just depend on the school and these teacher(s) and different organizations to teach your kids. You have to teach your kids, first” (*Black woman, Atlanta*).

Despite the recognition that women needed training in how to speak with their children, participants also noted children may not feel comfortable coming to adults when they become sexually active or curious and that it may be challenging for parents if/when the topic is raised. As stated by one participant, “sometimes it’s embarrassing to talk to your kids. It’s very hard” (*Hispanic woman, Bronx*). Thus, participants suggested that programs should teach women to address barriers to these conversations and assist women with overcoming feelings of embarrassment.

##### Multilevel empowerment

Rather than have a program that focuses solely addressing one aspect of women’s HIV risk (e.g., behavior), participants suggested addressing risk at multiple levels via empowering programs that include traditional clinical services (e.g., HIV testing and counseling) integrated with behavioral interventions and social services. For example, one participant stated that if she were creating an HIV prevention program, she would make sure it included, “home economics, sex education, business skills ... and social services” (*Black woman, Bronx*). Capturing the voices of numerous women, another participant outlined her vision of an ideal and comprehensive approach to prevention for women:

… Educational classes and help ... They … need help to finish school. They need help with childcare… helping these women with some cash and some food stamps. Help them with educational classes… give them the childcare that they need … Something … that would … have everyone feeling like they can turn there for help. Like the door is never shut or something.

*Black woman, Bronx*

In numerous groups, participants reinforced the need for programming to go beyond simply providing educational sexual health classes, which implied an understanding of HIV risk as multidimensional. A few participants also recognized the importance of layering prevention initiatives by first addressing women’s most pressing needs (e.g., food, shelter, financial security, and emotional wellbeing) and then moving on to more traditional approaches to HIV prevention that are interwoven with empowerment approaches. Providing an example, a Black woman from North Carolina, stated that programs should offer “regular testing [and] life planning counseling. So, she needs education first [then] empowerment and self-esteem reinforcement and testing… All that and prevention needs to be available.”

#### Create engaging program content

Across groups, approaches to HIV prevention were most often conceptualized as behavioral interventions. In this regard, participants discussed the need for behavioral interventions to employ participatory activities that encourage engagement. This recommendation often emerged as a disagreement in groups where a few participants initially suggested less engaging programmatic approaches such as having educational classes and distributing informational brochures. In numerous groups, participants responded to such suggestions by highlighting associated drawbacks and offered alternative approaches that could engage participants in experiential and visual learning activities to encourage knowledge acquisition and skill building. “I see what she’s saying, a brochure, but half the time somebody hands me a brochure I’m not gonna read it. I don’t care what it’s got on it… I want something hands on” (*Black woman, Raleigh*). Similarly, another participant stated, “If you give me a pamphlet – hey when I leave out that door everything you just said is going right in the trash versus me *seeing* it” (*Black woman, Washington, DC*).

Participants provided examples of a variety of visual and hands-on activities that programs could employ, including a) showing pictures of active STIs to incite fear and motivate engagement in prevention, b) showing videos of active STIs so that women were able to identify symptoms of infections (e.g., bumps, discharge, etc.) in themselves and their partners, c) engaging women in role play to better learn sexual communication skills, d) demonstrating condom use to ensure women know how to correctly use condoms, and e) facilitating interactions with PLWHA individuals to learn firsthand about their physical and psychological experiences with the virus.

I think I probably would have a program … [with a] demonstration of how to put a condom on. I mean, have a dildo or banana, whatever you want to use to show them the proper way of using a condom …

*Black woman, Washington, DC*

Participants discussed the utility of hands on engagement, emphasizing that fun activities can build women’s self-efficacy, which will allows women to take safety into their own hands rather than relying on partners. Women stated that this was especially important in the context of condom use, as condom use should not be the sole responsibility of male partners. One participant emphasized that women “… know how to use [condoms], because you don’t know – [he] may not know how to put it on, so you might just have to do it …” (*Hispanic woman, Bronx*). To this end, many participants stated that programs should include condom use demonstrations for both male *and* female condoms.

In expressing the need for more engaging programming, participants also discussed the importance of utilizing “motivational speakers [that will] encourage these women to go out here and do right, love themselves, and protect themselves” (*Black woman, North Carolina*). Many participants discussed the need for women to be exposed to the unique perspectives and experiences of HIV positive individuals to learn more about how individuals contracted the virus and live with HIV.

I’m all about … motivational speakers but they would have to be HIV positive people. Because only people who don’t look like they have it, who have it, can really show other women that you can’t tell who has it or not …They need to see that… They need to see women with HIV living day to day, men with HIV living day to day, living healthy productive lives …

*Black woman, Washington, DC*

Overall, participants expressed that there were no viable substitutes for being exposed to the perspectives of HIV positive facilitators. “You find someone that’s dealing with it, that’s living with it… don’t bring me somebody that’s doing research, because I can do the same research you’re doing” (*Black woman, Bronx*).

Although they mentioned the import of being able to hear these stories, they also highlighted that programs should include more dialogue versus presentations. Participants felt that women would be more engaged if women could talk with each other and the facilitators in a guided discussion format. In the words of a Black woman from the Bronx, “You need it to be interesting. It’s serious, but it shouldn’t just be something where you’re just sitting there, and they’re doing all the talking. No, everyone talks… It’s discussion.” In addition to ensuring that groups were discussion based, participants also stated that the groups should be small and start with fun icebreakers. Participants highlighted that the discussion groups could serve as sources of support for women and could also be a safe environment where they can talk about and receive help with their experiences with sex, condom use, sexual communication with partners and kids, relationship dynamics, drug and alcohol use, family planning, and domestic violence. Despite the seriousness of these topics, women stated the need to infuse the group discussion with fun and interactive activities to maintain engagement.

Some groups are dead. Nobody wants to sit there for an hour listening to: ‘what are STDs? … Can anybody tell me how you contract HIV?’ That sounds boring. I would be sitting there going, ‘Oh. My. God. When is lunch? … What are they giving out for free? Am I getting paid for this?’ I would literally be sitting there bored to death… But they need more talk groups…livelier talk groups.

*Black woman, Bronx*

Through this recommendation on *how* interventions should be delivered, participants suggested that discussion groups would honor women’s desire to be heard and feel their opinions are valued.

#### Build “market demand”

Participants discussed that prevention programs needed to employ marketing strategies to ensure interest and desirability in the target audience, emphasizing that programs needed “to make somebody’s mouth water for it” (*Black woman, Bronx*). Suggestions for stimulating interest in programming included employing media campaigns via targeted promotions and advertisements on television and in areas frequented by women such as restaurants, parks, bathrooms in bars, and hair salons. Relatedly, participants suggested having programs promoted by national and local celebrities. For example, a participant discussed the popularity and influence of a local radio personality and discussed how individuals with a platform and large audiences could quickly engage and attract large followings in prevention activities.

Let Big G put on a flyer ‘free admission [to the] Backyard [Band] show for HIV testing, bring your proof of HIV [testing]’. Girl you know that line [is] going to be like the Million Man March outside… Big G can come on the radio and be like ‘we got tacos and condoms’! … Let him … be like ‘HIV - that’s the new thing to do, go take a test.’ Everybody going to be in the DC area talking about ‘stick me right there’!

*Black woman, Washington, DC*

Participants also discussed the need for strategic incentivization as an approach for increasing market demand. Recommended incentives included food, giveaways, childcare, gift cards, cash, and vouchers for gas or public transportation. Participants also recommended including a variety of free condoms, “free HIV testing, free pap smears, [and] free medication” (*Black woman, Washington, DC*). Participant responses implied a desire to see free health services co-located with HIV prevention services and programming, suggesting a desire for improved access to health care. Relatedly, during a focus group exchange in the Bronx, three Black women stated,

“As long as it’s free you will be amazed at how fast people come.”; “People will come. Offer some food with it.”; “That is so true, sweetie.”; “As long as it’s free, you’d be amazed at how fast they’d come.”

In addition to suggesting free programming, some participants recommended paying individuals to engage in prevention activities.

Oh, you’ll pay me fifty dollars! Oh, I’ll go … and everybody’s like, girl I ain’t going to go get no AIDS test …I was like they pay you fifty dollars and then everybody [was] like ‘come on let’s sign up’ …

*Black woman, Atlanta*

Overall, participants acknowledged the importance of having high quality intervention content but also emphasized the necessity of designing and implementing high quality marketing strategies to stimulate interest and engagement in prevention activities. “Because you got to really … get these people’s attention nowadays. You got to really have a good, good, good, good idea – to get somebody to stop what they doing” (*Black woman, Washington DC*).

##### Branding

When participants were invited to provide their suggestions for a prevention program logo or motto, they mentioned that the visual appearance of the prevention approach was key for flyers and other branded materials. “It’s all in the presentation” (*Black woman, Bronx*). General discussion suggested that both the logo and the motto must be memorable, attractive, and mentally stimulating. “You need to reach their mentality [with a] little catchy slogan, you know. ‘Condoms Equal Life’ … a real message that can hit hard” (*Black woman, Washington DC*).

Participant dialogue suggested that marketing materials for programs, especially the logos, demonstrate unity, women, safety, and action. One participant discussed the importance of symbolizing action by stating, “It’s not even about being aware because everybody is pretty much aware now. You have to actually do it, not just say [it]” (*Black woman, Bronx*). Additionally, participants recommended the motto for the program to demonstrate safety, community, action, and consequences of risky sexual behavior. One participant simply said, “United we stand. That’s it” (*Hispanic woman, Bronx*)*,”* expressing the importance of community being demonstrated in the motto.

#### Ensure accessibility

Accessibility was also stressed as a key component of prevention programming. Participants suggested several strategies to increase access to prevention while eliminating barriers to engagement such as medical mistrust, stigma associated with accessing sexual health services, lack of awareness regarding where to access prevention resources, and transportation to program sites.

I would be more down to earth… have like an outreach program also, go around with this pack [of prevention materials] … because you know people are scared, they’re afraid… They don’t want you to know … that they’re buying in the stores, … they’re embarrassed by it … So, I would like an outreach program, like when the truck come out and pass out condoms.

*Black woman, Washington DC*

In addition to community-based outreach, other suggestions included having community members host and facilitate programs, implementing community-based “door to door” programming, employing peer education strategies, and engaging individuals in prevention through volunteerism.

Try to involve -- I think, try to involve the community. You know, try to go out there and … see if you can get volunteers, someone to actually go out and involve themselves… try to get people to come out and recruit. ‘Hey, let’s hand out fliers!’

*Hispanic woman, Bronx*

Several of the community-based strategies were also highlighted as approaches that could help participants feel safe, interested, and open to participating in prevention programming.

In order to ensure the greatest possible level of engagement and accessibility, it was recommended that community members be part of the program design team. “I would just make everything accessible for nothing. You know? Because it’ll make a person come on in. Design a program with folks – to bring women in” (*Black woman, Washington DC*)*.* Additionally, participants stated that combining community-based approaches with other types of approaches would be most effective in helping women engage in and access prevention programming as the target population would be repeatedly exposed to the same messages, information, and resources.

### Results of disconfirming case analysis

Our efforts to identify contrary cases in the data resulted in the following thematic results: 1) Address Structural Risk Factors (Subthemes: Reduce Poverty as a Risk Factor and Employ Policy to Mandate Prevention Participation); 2) Expand Awareness of and Access to Prevention Resources, and 3) Increase Engagement via Pleasure Promotion. The following paragraphs detail these findings.

#### Address structural risk factors

Empowering women economically and creating policies that mandated participation in prevention via testing and educational classes were suggested by a few participants as a strategy for improving prevention initiatives.

##### Reduce poverty as a risk factor

Results revealed recognition of a need for programs to address structural factors that impact HIV risk, including poverty. Some participants believed that equipping women with the skills needed to obtain gainful employment would help prevent women from engaging in risky behaviors, especially those who do so in an effort to meet their daily living needs. It was also acknowledged that before conversations can be had about safer sex, prevention programs need to first address women’s most pressing concerns, which are often related to meeting basic needs (i.e., food and shelter).

Most times, it’s finances, and women get themselves into a situation – they have to do it to feed their children … and they’ll take higher risks to get what has to get paid, so there [needs to be] more programs out to help them pay their bills, and feed their children, and clothe them.

*Black woman, Bronx*

Economic empowerment was viewed as a “way out” of the financial hardship that influenced some women to engage in risky behaviors to provide for themselves and their children. Describing these challenges and providing a roadmap for prevention programs, one woman explained,

If you sit there and tell me, okay, I understand that you’re having problems paying the bill and I understand you’re out here doing this, so how about I help you get into some job training ... I’ll find resources to help you better your situation so that you’re not struggling here and feeling like you need to take up slack with selling sex or end your depression by using drugs … I just would like them to offer some real resources that are reachable for women…

*Black woman, Raleigh*

In other words, empowering women with tangible support can facilitate women’s increased capacity to make choices they deem best for themselves via increased confidence and access to resources needed to manifest their desired behaviors and outcomes.

##### Employ policy to mandate prevention participation

Data analysis also revealed controversial suggestions for addressing social determinants of health via access to prevention programing and resources, including mandatory participation. A few proposals were offered in this regard. For example, a Hispanic woman from the Bronx simply recommended practitioners “make them mandatory sex education classes” and suggested this may be most useful for women with low incomes or those receiving public assistance. In addition, in a focus group exchange in the Bronx, a few participants agreed that participation in prevention initiatives should be mandatory.

“It’s mandatory that they have to go to this class. It’s by the law. I would want that to be in the program… We send you a letter, and you don’t come to this class, you get in trouble for it … you don’t want to pay no fine, so you better come to the class. And then we’ll make it fun … [but] I feel it has to be a mandatory class for everybody to go…”;

“That’s right.”; “Free but mandatory.”; “It’s mandatory by law that you show up.”; “Or you will not get no food stamps this week.”

*Three Black Women, Bronx*

#### Increase engagement via pleasure promotion

To create more engaging programmatic content some participants suggested teaching women strategies to increase sexual pleasure for themselves and their partners. In a focus group of Black women in the Bronx, one participant suggested teaching women how to perform oral sex for a male partner because “it’s is a good skill [to have].” Other participants in the same focus group agreed and discussed popular sex symbols in the media that provided examples on how to please their partners. Similarly, in another focus group in the Bronx with Hispanic women, participants discussed learning oral sex techniques in the context of safer sex promotion. One participant emphasized, “My point is … there should be … ways to make safe sex fun… knowing that you’re talking about your safety or whatever, maybe you find different ways to make it fun.”

A few participants suggested that sexual health programs should not exclusively focus on prevention but should comprehensively approach sexual wellness and include pleasure as a focal point in programming for women. Emphasizing the utility of this approach, a Black woman from Atlanta discussed pleasure-focused skills training as a strategy for increasing women’s interests in HIV prevention programming as well as an alternative to partnered sexual relations.

I might [get] some toys for them… [If] you can’t have sex, go ahead and play with yourselves… [I would give them] … sexual toys, like vibrators, dildos… [You can] put the babies to bed and go about your business.

#### Expand awareness of and access to prevention resources

Suggestions for increasing access included providing location-based text and online alerts to ensure maximal awareness of prevention initiatives and having services and facilities that provide continuous access to prevention resources, especially for high-risk populations.

My program would have websites. It would have a 24 hour phone line and it would tell you things like if you’re out in the street like if you’re a street worker, if you prostitute and stuff it’ll tell you the nearest location to find condoms, free condoms.

*Black woman, Washington DC*

Relatedly, participants recommended making free condoms available in locations that are easily accessible and open for longer hours such as local corner stores and gas stations.

I know sometimes you just run out or you short on supply or something… The free condoms, even though they’re free, they’re not available in every corner store. It’s not in walking distance… So I would say just, promote more condoms.

*Hispanic woman, Bronx*

One participant recounted an experience of visiting a local corner store that carried condoms.

I mean he had women’s condoms. He had men’s condoms. He had finger condoms… And since he tore down his store – in the hood, it’s not a lot of stores out here now that gives out condoms.

*Black woman, Raleigh*

This participant highlighted the convenient nature of being able to quickly and easily access condoms and the unfortunate nature of a limited number of stores offering free condoms, suggesting a desire for free condoms to be offered in more areas that are quickly accessible.

## Discussion

Despite recent successes in HIV prevention and treatment in the US, sustained epidemic rates of HIV infection among certain social groups indicate formidable barriers to HIV prevention [7–9]. Highlighting concerns around the ability of the US to meet national and international HIV prevention goals, the CDC has emphasized that effective prevention and treatment interventions are not reaching those most in need, especially those living in high HIV burden counties [3, 10, 18, 38]. By centering the voices of women living in four cities heavily impacted by HIV, this qualitative study outlines suggestions for developing new and improving existing HIV prevention programs. Women with high geo-behavioral and socioeconomic risk for HIV suggested specific program strategies to increase impact, engagement, and accessibility.

### Ensuring impact

To empower women at the individual level, participants called for promotion of self-esteem. This recommendation is supported by literature that identifies low self-esteem as a predictor of decreased self-efficacy for condom negotiation, increased perceived barriers to condom use, more negative condom attitudes, and higher HIV risk behaviors [39–42]. In addition, low self-esteem is related to psychological distress [43], another predictor of HIV risk behavior [44]. In light of these findings, scholars, like the participants in the current study, have called for self-esteem to be addressed in the design and adaptation of risk reduction and prevention programs.

Participants also stated that interpersonal or relational empowerment via training in sex communication skills (for partners and children) was necessary for programs and suggested that acquisition of these skills could be facilitated by increasing women’s self-esteem. Based on previous research [39], it is plausible that programs facilitating increases in self-esteem have potential to decrease risk at individual and interpersonal levels. In addition, although not mentioned by participants, risk reduction programs addressing interpersonal empowerment could also target couples and address issues related to intimate partner violence, substance use, sexual health communication, and HIV testing. While such programs are useful, programs that focus exclusively on individual and interpersonal behavior change often neglect community, structural, and other interrelated factors that influence behavior and impact risk [45].

In this regard, participants identified structural factors and subsequent behaviors that interact to impact HIV. For example, they highlighted that low socioeconomic status likely impacts decisions to engage in “survival sex” [11]. To address this issue, participants recommended addressing economic risk factors. Moreover, participants implicitly acknowledged that women’s risk simultaneously manifests at multiple levels and should thereby be simultaneously addressed at multiple levels. Indeed, researchers have characterized the nature of women’s risk for HIV as multifaceted [11, 45, 46]. However, intervention efforts for women principally remain unilevel [45, 47]. Such approaches highlight the deficiencies of individual level appraisals of HIV risk and limit potential macrolevel heath changes that can be achieved by implementing a package of multilevel and combination interventions [48, 49].

Though effective biomedical, community, individual, and interpersonal level interventions exist in the CDC’s Compendium of Evidence Based Interventions for HIV prevention [47], there remains a need for programs that *simultaneously* address *multilevel risk* among women. This need represents an opportunity for the field to develop multilevel programming and adapt existing evidence-based programs, particularly in high HIV burden counties. To this end, participants suggested developing empowering multilevel programs by integrating traditional clinical services (e.g., distribution of condoms and HIV testing) *with* behavioral interventions and social services. These findings emphasize the need to co-locate services to facilitate greater access to care, which may address underlying structural issues and disparities that further drive the HIV epidemic.

Relatedly, findings suggest that women do not have the capacity to negotiate safer sex if they are more concerned about where they will live, what they will eat, and/or whether or not their children have their needs met. Thus, prevention programs and initiatives may be most impactful if they first address social determinants of health before addressing interpersonal, behavioral, and psychological risk factors, which are primarily the focus of the majority of interventions for women [47]. Taken together, the aforementioned suggestions stand to improve the ability of health professionals to meet the unique HIV prevention needs of women, especially those of Black women, who represented the majority of our sample.

### Bolstering engagement

Implicitly underscoring lack of engagement as a barrier to effective implementation, “create engaging program content” and “build market demand” emerged as suggestions for prevention programs. Participants offered several suggestions that are currently utilized in HIV prevention campaigns and programs (e.g., Greater Than AIDS [50] and Sister to Sister [51]), including engaging national celebrities, incorporating visuals, incentivizing participation, employing various hand-on activities, and utilizing role play activities to capture interest and assist participants with retaining and applying intervention material [47]. Novel suggestions for increasing engagement, not often utilized in primary prevention programs, included involving PLWHA in prevention programs designed for HIV negative women and offering pleasure focused activities in programming (e.g., teaching women how to pleasure themselves or a partner versus simply eroticizing safer sex). Although these strategies are not frequently utilized in prevention programs for women, they demonstrate promise for effectiveness as they have been shown to reduce HIV stigma and increase preventive behaviors for other groups [52–55].

Another strategy recommended for growing responsiveness to programs included employing strategic and targeted marketing and branding strategies that reflect values of the target population. Although engagement and uptake are key implementation challenges for HIV prevention, the fields of medicine, public health, and social and behavioral sciences have lagged behind in ensuring that prevention programs and interventions are inclusive of comprehensive marketing strategies that facilitate engagement. Often an afterthought, this key aspect of implementation can assist with improving reach, engagement, and behaviors of the target population and acceptability and uptake of an intervention [56, 57].

Considering these challenges, participants suggested “meeting people where they are” and bringing prevention to them in familiar places via familiar channels. For example, participants suggested utilizing local celebrities (e.g., radio personalities, musical artists, etc.) with large local followings in key catchment areas to disseminate prevention messages and advertise programs. This approach, which can also be applied in the context of social media with “influencers” on Instagram and Facebook, can be an effective marketing strategy as it can facilitate quickly reaching the target audience as well as more rapid establishment of trust and increased awareness. Moreover, social marketing campaigns in HIV prevention have demonstrated effectiveness in increasing HIV testing and other prevention behaviors [56–58]. As an example, the Johns Hopkins Center for Communication Programs’ utilizes “Entertainment-Education” (also called edutainment”) via audio visual media, social media, social marketing, and strategic marketing and branding strategies to increase understanding of and engagement in HIV prevention, which has resulted in health promoted behaviors across varied target populatoins [59]. The suggestions provided can be used to inform comprehensive marketing strategies that spur uptake of and equitable recruitment and retention in interventions.

### Increasing accessibility

Participants also discussed the need for implementation of community-based intervention strategies (e.g., utilization of community health workers for door to door outreach) as well as the need to include the target population in the design of prevention strategies. Such strategies are increasingly being utilized and have been informed by community-based participatory and action research approaches. Within this theme, participants also offered a more novel recommendation to increase accessibility to prevention resources, in particular condoms, by making them available for free in venues that are closer to their homes, less stigmatized (e.g., not healthcare facilities), and frequented by the target population on a daily basis. Specifically, participants suggested offering free condoms in gas stations, corner stores, bar bathrooms, hair salons, lingerie stores, and clothing stores.

This recommendation speaks to the importance of availability and accessibility – both of which are in line with the CDC’s Strategic Planning Guidelines for Condom Distribution [60], an integrated (e.g., biopsychosocial) approach to prevention which also includes guidelines for ensuring condoms are acceptable by the target population. Although not mentioned in the current study, the suggestion to make condoms more available and accessible can also be relevant to Pre-exposure prophylaxis (PrEP), a desired prevention option among women in the US [61]. Despite PrEP’s high effectiveness rate of reducing risk of HIV transmission [62], it remains widely underutilized by women due, in part, to lack of awareness, stigma, and challenges with accessibility [61, 63, 64]. Increasing awareness of PrEP and integrating it with existing interventions and multilevel programming while simultaneously making it more accessible, available, and socially acceptable may result in increased uptake among women. Of note, our data were collected before PrEP became widely available in the US.

Another, more controversial, participant suggestion for ensuring accessibility to prevention resources was to legally mandate engagement. Although the US has not adopted any universal prevention mandates, there is precedent for routinized screening. For example, HIV testing is required for pregnant women in some states and several states utilize “opt out” testing approaches [65], in which HIV tests are conducted along with other standard screenings during pregnancy unless the woman opts out. Such policies can result in testing increases for pregnant women, including increasing rates of testing among pregnant women up to 98% [65].

Other countries have executed more concentrated national policies to prevent HIV and mandate engagement in prevention. For example, Cuba, designated by the World Health Organization as the first country to eliminate mother to child transmission of HIV, requires testing for all pregnant women and individuals who test positive for other STIs [66, 67]. These policies have contributed to Cuba being among countries with the lowest prevalence rates in the world [68, 69]. Although these policies would likely be extremely controversial in the US, it is possible that employing opt out testing for certain populations (e.g., pregnant women and those who test positive for other STIs) may be more acceptable and feasible and could help contribute to meeting goals of the National AIDS Strategy [1, 2].

### Limitations

While our sub-study sample is not representative of all US women at HIV acquisition risk, it is important to note that the overall HPTN 064 cohort was at demonstrated increased risk for HIV acquisition. One limitation is the age of the data, which were collected 10 years ago. It is possible that participants may have provided different responses if the data had been collected more recently. Another limitation was the lack of a connection between detailed participant demographic data (e.g., SES, age, or relationship status) and participant qualitative data. Having these data connected could have allowed us to better contextualize participant responses and make comparisons among participants based on demographic characteristics. Despite these limitations, the current analysis of the subjective opinions of selected participants provides substantive information to researchers and interventionists seeking to create or improve HIV prevention programs and initiatives. While our analysis included only a portion of transcripts from the overall qualitative data gathered in the larger study (i.e., 30% of the interview transcripts and approximately 50% of the focus group transcripts), the number of transcripts reviewed was based on principles of data saturation [32].

## Conclusion

We have the information and tools needed to end HIV but have not yet done so [3]. The suggestions presented herein bear relevance for development and implementation of behavioral and biobehavioral interventions for racially, ethnically, and socioeconomically marginalized women which may contribute toward the advancement health equity in HIV prevention. As we learn more about the multidimensional nature of women’s risk for HIV, there are increasing opportunities to utilize suggestions from the target population to better understand and overcome barriers to optimal sexual health and to develop and promote gender relevant interventions, especially for locations with great need for expanded and improved prevention services. As highlighted by participants, existing prevention programs have employed useful strategies but there is room for improvement. Our findings situated in the context of existing research suggest that lending increased attention to empowerment, marketing, and accessibility has potential to result in positive outcomes for HIV prevention programming. Moreover, the data presented herein provide a platform for the voices of the target population, voices often not heard, which fills gaps in the current literature and provides critical insights to support bringing the plan of *Ending the HIV Epidemic*^18^ closer to reality.

## Data Availability

Available upon request from the HIV Prevention Trials Network who serves as the data coordinating center and repository of record for this study. The corresponding author can assist with facilitating this request.

## Abbreviations

US: United States
HIV: Human Immunodeficiency Virus
AIDS: Acquired Immunodeficiency Syndrome
PLWHA: People Living with HIV/AIDS
HPTN: HIV Prevention Trials Network
DC: District of Columbia
USD: United States Dollar
ICR: Intercoder Reliability
STD: Sexually Transmitted Disease
CDC: Centers for Disease Control and Prevention
PrEP: Pre-Exposure Prophylaxis
